# At-line determining spore germination of *Penicillium chrysogenum* bioprocesses in complex media

**DOI:** 10.1007/s00253-016-7787-y

**Published:** 2016-08-24

**Authors:** Daniela Ehgartner, Jens Fricke, Andreas Schröder, Christoph Herwig

**Affiliations:** 1 CD Laboratory on Mechanistic and Physiological Methods for Improved Bioprocesses, Vienna University of Technology, Gumpendorferstrasse 1a/166, 1060 Vienna, Austria; 2 Institute of Chemical Engineering, Research Area Biochemical Engineering, Vienna University of Technology, Gumpendorferstrasse 1a/166, 1060 Vienna, Austria

**Keywords:** Filamentous fungi, Flow cytometry, Spore germination, Spore viability, Process analytical technology

## Abstract

Spore inoculum quality in filamentous bioprocesses is a critical parameter associated with viable spore concentration (1) and spore germination (2). It influences pellet morphology and, consequently, process performance. The state-of-the-art method to measure viable spore concentration is tedious, associated with significant inherent bias, and not applicable in real-time. Therefore, it is not usable as process analytical technology (PAT). Spore germination has so far been monitored using image analysis, which is hampered by complex medium background often observed in filamentous bioprocesses. The method presented here is based on the combination of viability staining and large-particle flow cytometry which enables measurements in real-time and hence aims to be applicable as a PAT tool. It is compatible with the complex media background and allows the quantification of metabolically active spores and the monitoring of spore germination. A distinction of germinated spores and not germinated spores was based on logistic regression, using multiparameteric data from flow cytometry. In a first step, a significant correlation between colony-forming unit (CFU) counts and viable spore concentration (1) in an industrially relevant model bioprocess was found. Spore germination (2) was followed over the initial process phase with close temporal resolution. The validation of the method showed an error below 5 %. Differences in spore germination for various spore inocula ages and spore inoculum concentrations were monitored. The real-time applicability of the method suggests the implementation as a PAT tool in filamentous bioprocesses.

**Electronic supplementary material:**

The online version of this article (doi:10.1007/s00253-016-7787-y) contains supplementary material, which is available to authorized users.

## Introduction

Spore inoculum quality in filamentous bioprocesses is a critical parameter associated with viable spore concentration and spore germination (Nielsen and Krabben 1995). It influences pellet morphology and, consequently, process performance (Paul et al. 1993; Smith and Calam 1980; Tucker and Thomas 1994). An essential step before inoculation is the determination of the viable spore concentration in order to apply quality control and decrease batch-to-batch variability. The state-of-the-art method to investigate this variable is colony-forming unit (CFU) determination, which assesses the number of viable/germinating spores. This procedure is tedious, associated with significant inherent bias, and not applicable in real-time.

Previously, an at-line method based on a flow cytometer (FCM) and viability staining to investigate the concentration of viable spores has been developed. This method is based on the metabolic activity of viable spores and showed a correlation to CFU (Ehgartner et al. 2016). But, also spore germination is an important factor linked to spore quality. Germination is especially crucial in the CFU method, as only spores growing hyphae can form colonies. Therefore, monitoring of spore germination is not only necessary to investigate further spore quality attributes, but can also be used as a validation of the aforementioned method. Quality attributes connected to spore germination are not only the amount of germinating spores but also how long spores need for germination and whether all spores germinate at the same time or not. Spore swelling and spore germination have so far been monitored using microscopy (Demming et al. 2011; Oh 1996; Paul et al. 1993), which is hampered by complex medium background often observed in filamentous bioprocesses (Posch et al. 2012).

Germination was reported to be influenced by a variety of parameters such as spore age (Paul et al. 1993), medium composition (Demming et al. 2011; Fletcher and Morton 1970; Paul et al. 1993), spore concentration (Demming et al. 2011; Fletcher and Morton 1970), temperature (Demming et al. 2011), pH (Demming et al. 2011) and storage conditions of the spore inoculum (Gottlieb 1950).

Fluorescence stains indicating metabolic activity in the cell showed to reflect transitions to other physiological and morphological phases (Bradner and Nevalainen 2003; Dorsey et al. 1989). Metabolic activity during germination in filamentous fungi was reported to increase (Gottlieb 1950). Fluorescein diacetate (FDA) is converted to a fluorescent product (fluorescein) via esterases and hence is a stain marking metabolic activity in cells (Rotman and Papermaster 1966). Therefore, it could be applied not only to stain viable cells (Ehgartner et al. 2016) but also to indicate spore germination.

The study described here is based on advanced flow cytometry, providing multiple data points per channel per particle. This signal or so-called pulse shape is achieved for both scatter channels as well as green, orange and red fluorescence channels. Common flow cytometers provide only one value (integrated signal) each for forward scatter (FWS), sideward scatter (SWS) and fluorescence channels for each particle (Díaz et al. 2010; Hyka et al. 2013; Pereira and Ebecken 2011; Rieseberg et al. 2001). So far, this additional information provided by the pulse shape has been used in an aquatic environment (Pereira and Ebecken 2011; Thyssen et al. 2011) and for urine particles (Delanghe et al. 2000). Pulse shapes give access to additional morphologic information of the cell and therefore could be applied to detect spore germination.

The feature of image acquisition in the flow cytometer facilitates method development and method validation. Thereby, flow cytometric data is connected to a picture of the cell. This picture taken in the flow cell replaces a separate microscopic investigation and has the advantage of being usable in higher throughput (George et al. 2004; Pereira and Ebecken 2011).

The goal of this study is to develop a tool to at-line monitor spore germination of *Penicillium chrysogenum* in the bioreactor. Therefore, a combination of viability staining and advanced flow cytometry is used. The viability stain FDA should be applied to distinguish viable spores from other spore sub-populations and complex media background. Furthermore, data from pulse shapes of fluorescence and light scatters is used to distinguish non-germinated and germinated spores. Thereby, a tool applicable at-line and which hence is adaptable as process analytical technology (PAT) should be provided.

## Material and Methods

### Strain

Spore suspensions of the P-14 *P. chrysogenum* candidate strain for penicillin production descending from the P-2 *P. chrysogenum* candidate strain (American Type Culture Collection with the access number ATCC 48271) (Lein 1986) were provided by Sandoz GmbH (Kundl, Austria) and used for all experiments.

### Bioreactor cultivations

Bioreactor cultivations were conducted in 10 and 20 l Techfors reactors (Infors, Bottmingen, Switzerland).

Fermentation temperature was kept at 25 °C via cooling/heating jacket. Aeration was controlled at 1 vvm in batch with mass flow controllers (Vögtlin, Aesch, Switzerland). Dissolved oxygen concentration was measured using a dissolved oxygen probe (Hamilton, Bonaduz, Switzerland) and maintained between 40 and 90 % by adjustment of the stirrer speed. The initial stirring speed was 320 rpm and the pressure was constantly held at 1 bar. pH was measured using a pH probe (Hamilton, Bonaduz, Switzerland).

The cultivations were carried out in batch mode on a complex bioreactor medium similar as described elsewhere (Posch et al. 2012). The pH was not controlled. The end of the batch was defined as an increase in pH of 0.5 by convention.

The culture was inoculated with 2* 10^8^–2 * 10^9^ spores/l cultivation medium. Spores of different ages (2–9 months) were used.

### Shake flask cultivations

Shake flask cultivations were conducted at 25 °C and 300 rpm in a Multitron Shaker (Infors, Bottmingen, Switzerland) on the same complex medium as used for bioreactor cultivations (but without antifoam). Five hundred-milliliter shake flasks were filled with 30 ml of sterile medium and were inoculated with 2 * 10^9^ spores/l cultivation medium.

### Sample preparation

Samples from shake flasks or bioreactor were diluted 1:10 into phosphate-buffered saline (50 g/l of 2.65 g/l CaCl_2_ solution, 0.2 g/l KCl, 0.2 g/l KH_2_PO_4_, 0.1 g/l MgCl * 6 H_2_O, 8 g/l NaCl and 0.764 g/l Na_2_HPO_4_ + 2 H_2_O) and stained with PI (Sigma Aldrich, St. Louis, Missouri/USA; 20 mM stock dissolved in DMSO, diluted to a final concentration of 20 μM) and FDA (Sigma Aldrich, St. Louis, Missouri, USA; stock solution of 5 g/l dissolved in acetone) was added to a final concentration of 50 mg/l. After an incubation of 10 min, the sample was further diluted (1:50 in the same buffer) for flow cytometric analysis.

### CFU determination

For CFU determination, the shake flask samples were diluted in a sterile solution of 8.5 g/l sodium chloride and 1 ml/l Tween 80 and plated on agar plates. The medium of the latter resembled the one used for shake flask cultivations and included 25 g/l agar agar.

### Flow cytometry

A CytoSense flow cytometer (CytoBuoy, Woerden, Netherlands) with two FWS, one SWS and three fluorescence channels (yellow, orange, red) was used for single-cell analysis. The implemented laser had a wavelength of 488 nm. The configuration of the filter set was 515–562 ± 5 nm for the green/yellow fluorescence channel (used for FDA) and 605–720 ± 5 nm for the red fluorescence channel (used for PI). The device was equipped with a PixeLINK PL-B741 1.3MP monochrome camera for in flow image acquisition. For data treatment, the software CytoClus (CytoBuoy, Woerden, Netherlands) and a custom-programmed Matlab 2009a script (MathWorks, Nattick, Massachusetts, USA) were used.

## Results

### Viable spore determination

In order to distinguish FDA positive (metabolically active/viable) spores from other spore sub-populations such as dead spores (see Ehgartner et al. (2016) for more detailed information) and media background, gate setting was conducted. Gates/boarders were set by measuring spores cultivated in filtrated medium, microwave treated spores and complex medium without spores in order to achieve a good distinction.

**Fig. 1:**
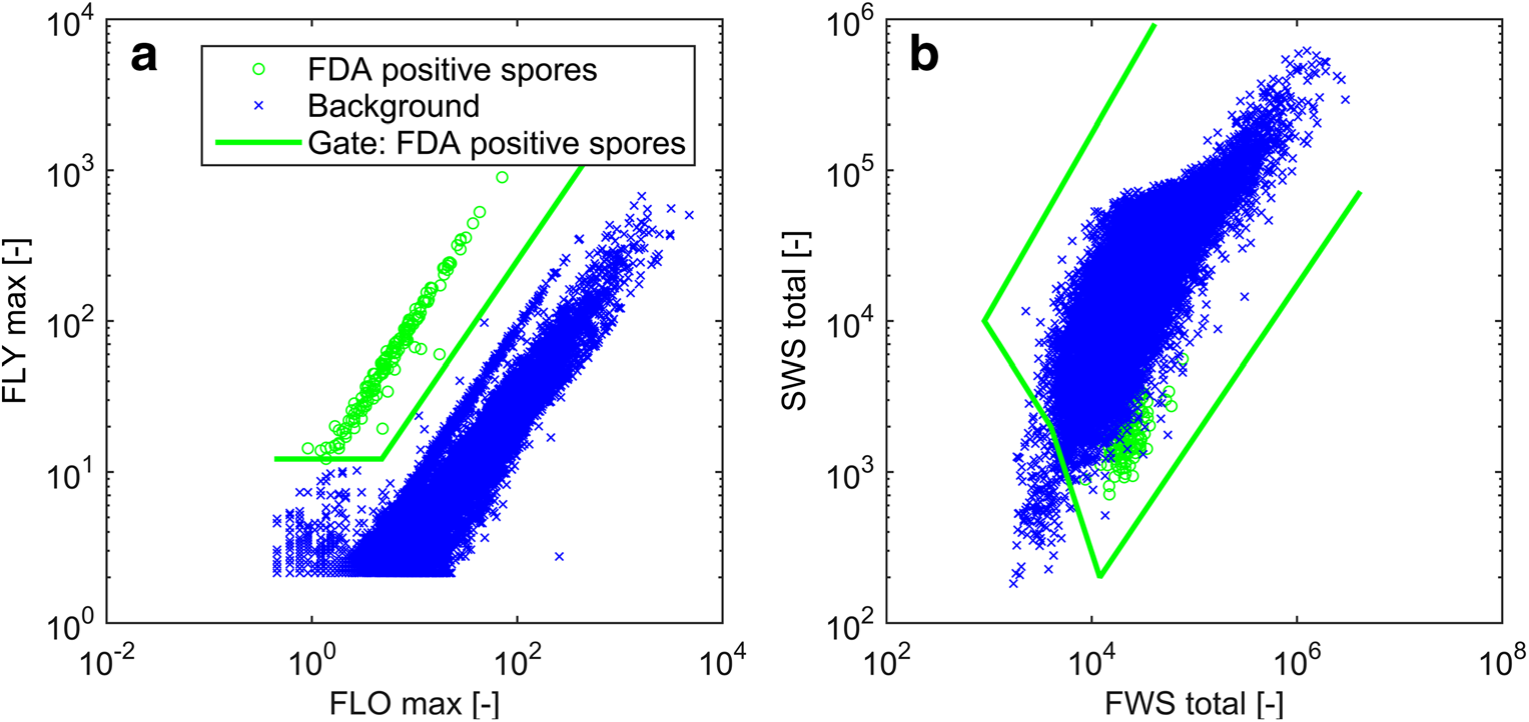
Gate setting to discriminate FDA-positive spores (*green circles*) from media background and other spore sub-populations (*blue crosses*). The classification was done via intensities in FLY and FLO max (**a**) as well as via FWS and SWS total (**b**)

The discrimination of these FDA positive spores from other particles was based on maximum (max) fluorescence yellow (FLY) and fluorescence orange (FLO) as well as on FWS total and SWS total, as shown in Fig. 1. FLY and FLO were applied to distinguish green fluorescent from not or red fluorescent spores and media particles. FWS and SWS were used in addition to exclude any green fluorescent particles smaller than the spores. Only particles found in both gates (in-between the green boarders in Fig. 1a, b) were classified as FDA-positive spores, while particles found in one but not the other gate were not counted. The distinction of these spores from other particles enabled the quantification of this population.

**Fig. 2:**
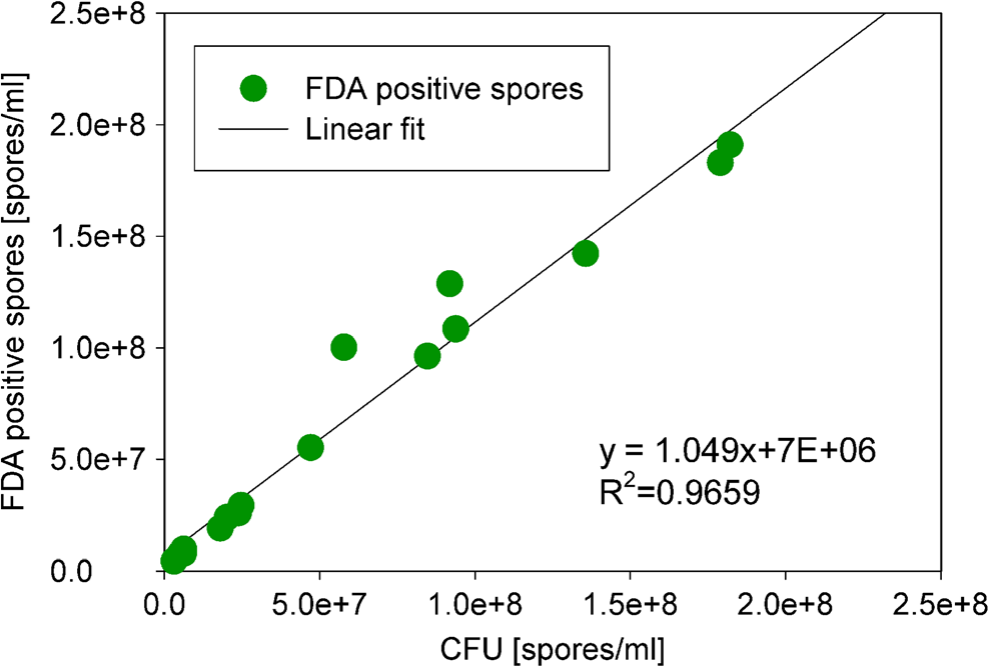
Correlation of FDA-positive spores and CFU counts. The slope of the linear fit was almost 1 with an *R* ^2^ of 0.97

Various spore inocula were measured with the FCM after 1 h of shake flask cultivation and the FDA-positive spore population was quantified. The same inocula (without previous cultivation) were platted on agar to result in a CFU count. The correlation of these showed to have a slope of almost 1 and an *R*^2^ of 0.97 (see Fig. 2). This demonstrates that the FCM method determines the amount of spores which germinate on agar plates and hence are defined as viable.

### Distinguishing germinated from non-germinated spores

**Fig. 3:**
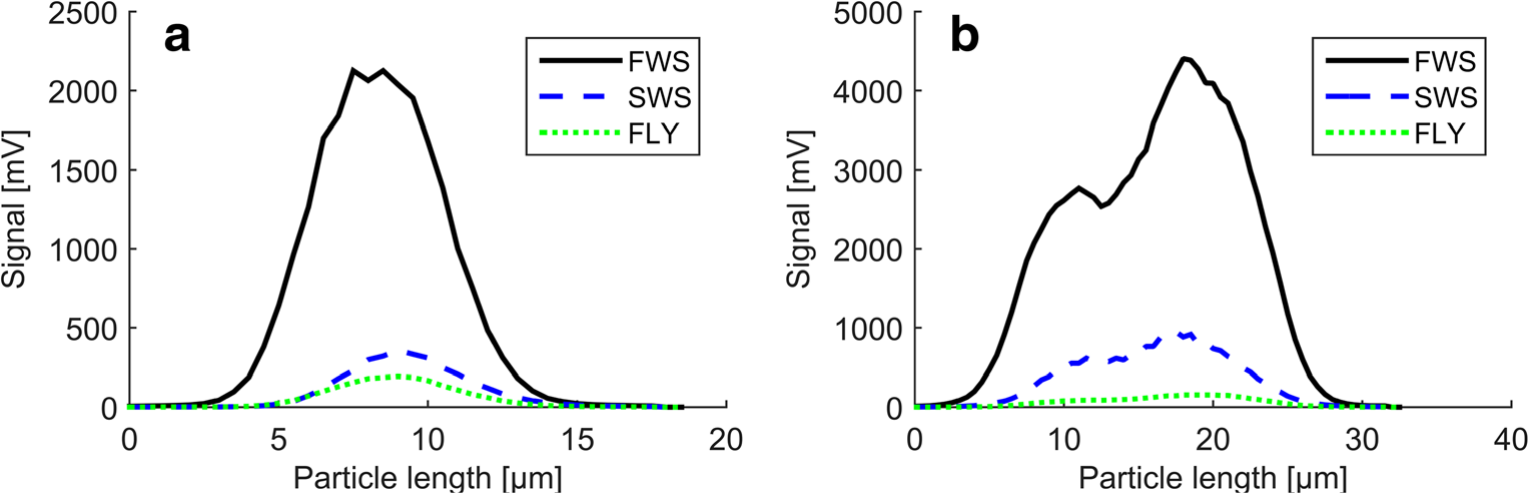
FWS, SWS and FLY signals of a non-germinated (**a**) and a germinated spore (**b**)

The FCM applied here provides multiple data points per channel and particle, the so-called pulse shapes. These pulse shapes differ for round (Fig. 3a) and germinated spores (Fig. 3b).

**Fig. 4:**
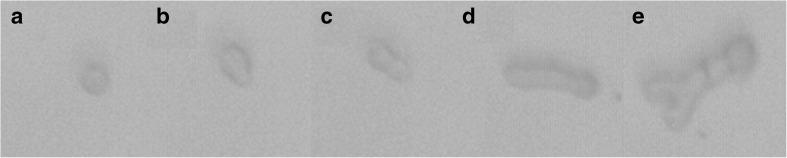
Spores and hyphae in the flow cell. **a** Non-germinated spore. **b** Germinating spore. **c** Germinated spore. **d** Unbranched hyphae. **e** Branched hyphae

Furthermore, the FCM makes it possible to photograph particles in the flow cell and to connect these pictures to FCM data. This feature was applied to create a data set allowing a statistical-based distinction of germinated and non-germinated spores. Different states of germination were distinguished on pictures as shown in Fig. 4.

According to literature, a spore can be defined as germinated when the germ tube has the length of one-half of the biggest dimension of the spore (Paul et al. 1993). In the study conducted here, spores as shown in Fig. 4a were defined to be non-germinated. Figure 4a represents a germinating spore which is in a state between being non-germinated and germinated. However, spores like the aforementioned one were determined to belong to the group of germinated spores. Spores resembling the ones in Fig. 4c–e were categorized as germinated.

In the fast particle flow through the system, spores and hyphae are (almost) always aligned the same way in the flow cell. The flow direction in Fig. 4 was from right to left. Obviously, the spores are oriented into the direction of the flow.

A logistic regression was applied on the data set based on shake flask samples, following the goal to distinguish the non-germinated spores as categorized before from the germinated ones, using data from FWS, SWS and fluorescence channels. The logistic regression revealed the following parameters to have a significant influence (*p* < 0.05) on the distinction of non-germinated and germinated spores: FWS length (length of the FWS signal in μm), FLY max (maximum of FLY signal), FLY inertia, FLY fill factor (indicates the solidity of the signal) and the coefficient of variation of the root mean squared error (CVRMSE) (also see Eq. 1 and Table 1). The latter parameter was calculated in Matlab (MathWorks, Nattick, Massachusetts, USA). Therefore, a Gaussian curve was fitted to the FWS signal and the CVRSME was applied as a quality factor of the fit. The other parameters were determined via CytoClus (CytoBuoy, Woerden, Netherlands). FLY inertia describes where the majority of the area beneath the curve is located in reference to the signal. Low inertia shows that a big part of the area below the signal curve is located near the centre of the signal curve. High inertia values indicate that a larger part of the signal area is near the edges of the signal.

1 $$ \ln \frac{p\left(\mathrm{n}\mathrm{o}\mathrm{n}\hbox{-} \mathrm{germinated}\;\mathrm{spore}\right)}{p\left(\mathrm{germinated}\;\mathrm{spore}\right)}={a}_0+{a}_1*FWS\;\mathrm{length}+{a}_2*\mathrm{CVRSME}+{a}_3*\mathrm{FLY} \max +{a}_4*\mathrm{FLY}\;\mathrm{inertia}+{a}_5*\mathrm{FLY}\;\mathrm{fill}\;\mathrm{factor} $$

**Table 1:** Estimated coefficients for two sensitivity levels

	FLY sensitivity level of 50	FLY sensitivity level of 65
*a*_0_	28.31	29.01
*a*_1_	−0.337	−0.346
*a*_2_	−0.184	−0.106
*a*_3_	−0.006	−0.001
*a*_4_	54.05	40.35
*a*_5_	−59.54	−52.44

The coefficients in Eq. 1 are similar for different sensitivity levels (see Table 1), especially concerning the FWS. Different levels of sensitivities of FLY had to be used for measurements over process time in order to extract the highest amount of information possible from each sample. During the first 12–16 h, a higher sensitivity was needed than later, during spore germination, when metabolic activity was high and would hence have resulted in a saturated signal.

### Validation via image in flow

The method was applied on samples after 14, 20 and 24 h of bioreactor cultivation, which represented different stages in the germination process. After 14 h of cultivation, the spores were swollen, but not yet germinated. After 20 h of cultivation, germination took place. After 24 h, the majority of spores was germinated and a high amount of unbranched hyphae and some branched hyphae were already found.

In order to validate the method, photographs from the flow cell were used to classify spores manually. By comparing the classification based on photographs with the classification via logistic regression, the method was evaluated. Table 2 shows the results of the evaluation for the measurement of FLY sensitivity level 65. The results were similar for the FLY sensitivity level 50 and are thus not shown.

**Table 2:** Contingency table for the evaluation of the spore germination method for samples measured at FLY sensitivity level 65

Cultivation time [h]	TP	FP	TN	FN	Total
14	0	1	134	0	135
20	114	2	37	20	173
24	124	1	6	4	135

Shown are the numbers of true positive (TP, germinated spores which were classified as germinated spores), false positive (FP, germinated spores not categorized as germinated), true negative (TN, non-germinated spores categorized as non-germinated) and false negative (FN, non-germinated spores categorized as germinated). Furthermore, the total amount of investigated spores is shown. Samples of three different cultivation times were evaluated

Comparing the photos from the flow cell with the classification based on the logistic regression, weaknesses of the method were revealed:

- 3.2 % of the non-germinated spores were wrongly classified as germinated in cases when there were particles from the medium sticking on the spore or when a media particle was very close to the spore while passing the flow cell (see Fig. S1a in the supplementary material).
- Germinated spores and unbranched hyphae which flow through the cell upright were wrongly classified as non-germinated (see Fig. S1 in the supplementary material compared to the bottom photo in Fig. 3). This is caused by the fact that spores floating upright through the flow cell have a FWS signal similar to a Gaussian curve. On average 2.9 % of germinated spore were wrongly determined to be germinated due to different alignment.

As stated above, germinating spores are seen to be in a state between being non-germinated and germinated spores. For method development this sub-population was categorized as germinated spores. If the allocation of germinating spores to both classes—non-germinated and germinated—was accepted, the wrong negative counts in Table 2 would decrease significantly. The errors of the method for different sampling points during the cultivation were calculated without considering the classification of these spores being in-between non-germinated and germinated. These are shown in Table 3.

**Table 3:** Error of the method to monitor spore germination

Cultivation time [h]	Error [%]	
	Sensitivity level 50	Sensitivity level 65
14	4.2	0.7
20	2.6	7.5
24	3.7	3.7

The error rate of 7.5 % in the 20-h sample when measured at a FLY sensitivity level of 65 underlines the importance to choose the right sensitivity level. During germination the metabolic activity in the spores is high. Therefore, the FLY signal is saturated leading to a wrong FLY max. Although FLY is often saturated when germination is finished, the classification into non-germinated vs. germinated spores has proven to be better. This is based on the increase of the hyphae length which further decreases the CVRMSE. In cases where the right sensitivity level had been chosen, the error of classification was below 5 %.

### Application of the method in bioreactor cultivations

#### Size distribution and distribution of green fluorescence over process time

**Fig. 5:**
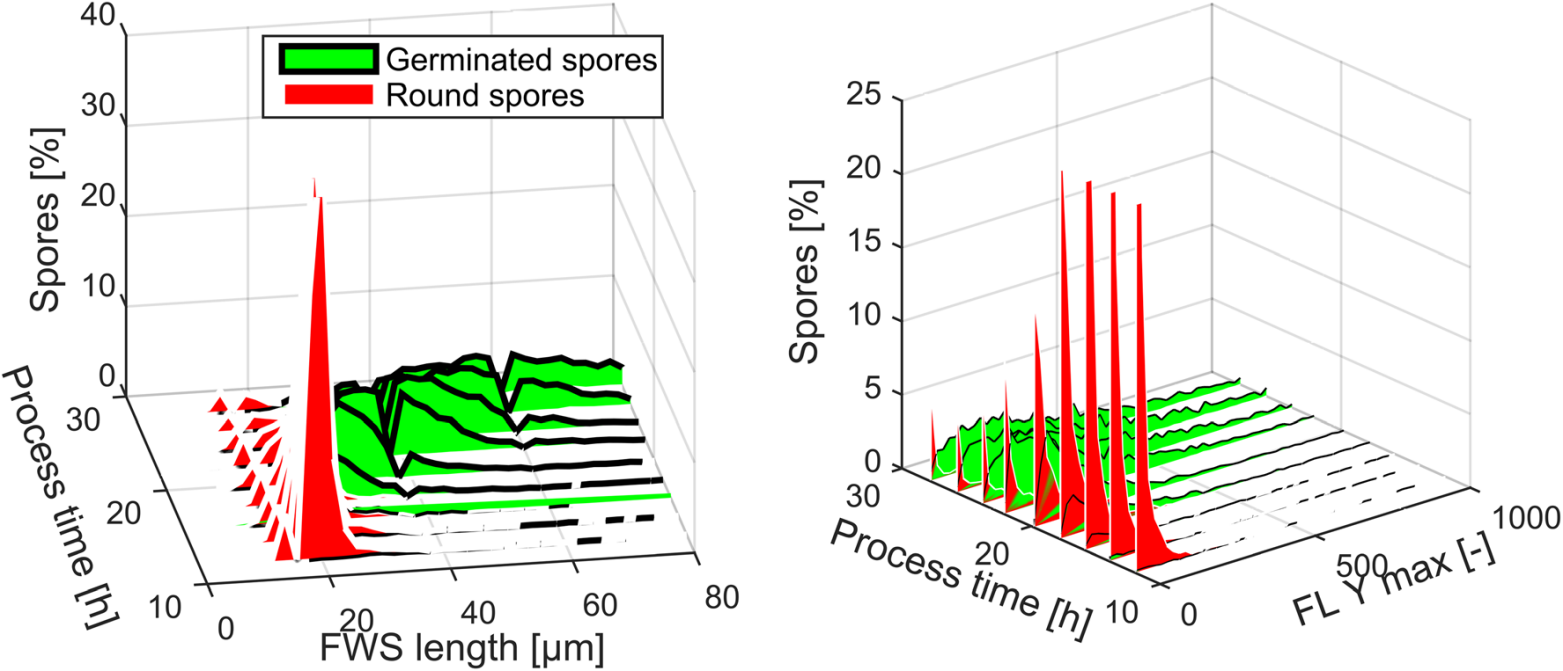
Size distribution (**a**) and distribution of FLY (**b**) of round and germinated spores in batch 1. The number of spores is normalized to show the amount of spores per sampling point in percent

The developed method to classify spore germination was applied on the FDA-positive spores in bioreactor cultivations. Total spore size (measured via FWS) and green fluorescence increased over process time. At approximately 25-μm spore diameter, the spores germinated as the transition of round to germinated spores in Fig. 5 shows. Furthermore, the FLY (esterase activity) increased fast during and after spore germination.

In addition to the FWS curve, these changes in spore size and green fluorescence are the signals used for the distinction of non-germinated and germinated spores.

#### At-line monitoring of spore germination

**Fig. 6:**
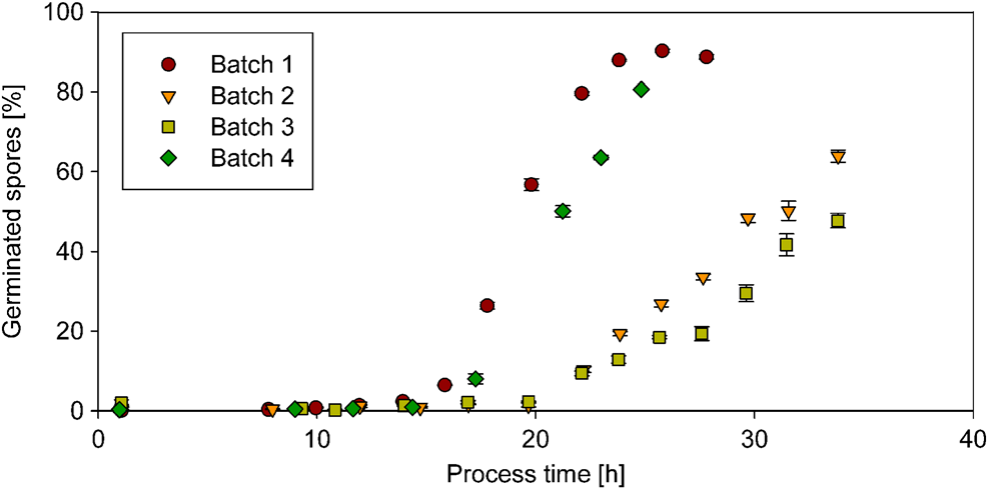
Spore germination over process time as percentage of total FDA-positive spore count. In batch 1 and 4, 90 % of the spores germinated. In the other two batches, the amount of germinated spores does not exceed 70 %

Spore germination was monitored at-line for FDA positive spores in batch cultivations in the bioreactor. For these experiments spores of different ages and different spore inoculum concentrations (2 * 10^9^ spores/l in batch 1, 2 * 10^8^ spores/l in batches 2–4) were used. The spore inoculum concentrations were determined beforehand with the method presented here using samples after 1 h of shake flask culture. At the beginning, no spores were germinated. After 15 h cultivation, germination started and within 10 h after the start of germination, the majority of the spores were germinated when younger spores (3 months, batches 1 and 4) were used. For older spore inocula (older than 8 months, batches 2 and 3), germination took longer and after 35 h of cultivation the percentage of germinated spores was lower than for younger spore inoculum after 20 h (see Fig. 6). The differences of spore germination between different spore inoculum concentrations were minor (compare batch 1 and 4 in Fig. 6).

Not all FDA-positive spores germinated. For spore inocula older than 8 months, the percentage of germinating spores was 60 % and lower. This is worth noticing as the correlation of FDA-positive spores to the CFU was almost one (also including measurements of these spore inocula). Therefore, it seems that spores germinate on agar plates, but not in a liquid environment in the bioreactor, although the media composition was similar.

## Discussion

### The necessity of at-line measuring spore germination

The method developed here, enabling the at-line determination of spore inoculum quality, revealed that not all spores determined as viable by state-of-the-art methods (CFU or combination flow cytometry/viability staining) do really germinate. According to the definition of viable spores made by Nielsen and Krabben (1995), only spores which germinate are viable. Based on this definition, neither CFU nor the flow cytometry/viability staining method proved to be suitable for the determination of viable spores for bioreactor cultivations.

These results underline differences of spore germination in liquid cultures and on agar plates. Spores which seem to germinate on agar plates do not germinate in liquid environment. Although the spores show metabolic activity and even swell (data not shown), they do not germinate. The same observation has already been made in previous studies (Paul and Thomas 1998).

Altogether, if a certain concentration of germinating spores is to be achieved in the bioreactor, the measurement of spore viability via CFU or flow cytometry is not sufficient. Therefore, two alternative procedures are suggested: first, a predetermination of the amount of spores needed for an inoculation can be carried out by shake flask cultivations. Spore germination can be evaluated in these flasks with the here presented method. Afterwards, the bioreactor could be exactly inoculated with the required volume of spore inoculum based on the spore germination investigation. A second possibility is to monitor spore germination in the bioreactor; this means that certain actions concerning parameters in the bioprocess can be set as a response directly to the at-line determined amount of germinating spores and the germination behaviour in order to improve the process performance.

### Advantages of the method developed here in comparison to previous ones

Spore swelling and spore germination have so far been monitored using microscopy approaches (Demming et al. 2011; Oh 1996; Paul et al. 1993). But even if automated image analysis was used—Oh (1996) and Paul et al. (1993)—microscopy is more time-consuming than flow cytometry, especially if high numbers of spores are to be investigated in order to increase the robustness of the result. But, the big benefit of flow cytometry in comparison to microscopy is its applicability in complex medium environment. Via fluorescence and scatter measurements, flow cytometry enables the distinction of spores even in highly complex medium, as shown in the present study and in Ehgartner et al. (2016). In contrast to this, spores on microscopy slides are covered by medium particles and therefore not accessible by the method.

By monitoring spore germination, it is not only possible to investigate germination kinetics, but also to get information about the total spore viability. Thereby, the state-of-the-art method CFU cannot only be replaced by the at-line flow cytometry; moreover, the here presented approach highlights the differences between fungal growth in liquid environment and on agar.

The time-resolved measurement of germinating spore concentration is an addition to spore quality measurements presented in Ehgartner et al. (2016). The decision tree described in the latter can be complemented by a further step—the spore germination. This leads to a separation of the viable sub-population in Ehgartner et al. (2016) further into a metabolically active but not germinating (hence, not viable) and a metabolically active and germinating (hence viable) sub-population.

### Applicability of the method in a process environment

The method presented here has been developed as an at-line tool, providing information in real-time. Therefore, it could be applied as a PAT tool (Rathore et al., 2010). Furthermore, flow cytometry is a method which is applicable online and which has been used in biotechnological processes (Hyka et al. 2013). In addition, the device used in this study (CytoBuoy, Woerden, Netherlands) is already applied in a fully automated way in aquatic environments (Malkassian et al. 2011). The online application of the tool developed here with enhanced automation would need some improvements. A big issue for invasive online tools such as flow cytometry is the sterile withdrawal of a representative sample at the bioreactor interface. Therefore, a flow injection system could be applied for sample taking and sample preparation, as it has already been described elsewhere (Broger et al. 2011; Zhao et al. 1999).

### Method transfer to other media and new strains

The method was shown to be applicable to a penicillin-producing *P. chrysogenum* strain. If the method is to be applied on other strains/species or another medium environment, two steps need to be completed beforehand. First, the viability staining has to be investigated on spores and media particles. Media particles should not be stained with FDA, neither should dead spores (after microwave treatment, for example). Spores from cultivation should largely fluoresce with FDA. In a second step, different spore inocula ought to be measured to do the correlation to CFU. This is important in order to confirm the staining and measurement procedure—the FDA concentration and FDA incubation time could influence which particles are determined as FDA positive and which are not.

The evaluation of the method to discriminate germinated vs. non-germinated spores is not necessary for new strains and is totally independent of the medium. However, adjustments of measurement parameters (e.g., sensitivity) in the flow cytometer software lead to a re-evaluation of the method.

### Benefits of the method developed here

To sum it up, the tool based on flow cytometry and viability staining is able to distinguish non-germinated from germinated spores in complex media environment. Spore germination can be monitored with an error rate of less than 5 %.

Not all spores germinating on agar plates also germinate in the bioreactor. There seem to be differences between solid and liquid growth. Therefore, measurement of spore germination is crucial to determinate the viable (= germinating) spore concentration in a bioreactor environment.

The implementation of this method as a PAT tool for at-line spore germination monitoring enables an intervention in early phases of the process. This offers a great opportunity in view of avoiding batch-to-batch variability. Furthermore, the monitoring of viable spores and spore germination on multiple processes and the linkage of these parameters to physiology and morphology in later phases enhances process knowledge.

## Electronic supplementary material

ESM 1 (PDF 69 kb)
