# Supplementary material for: At-line determining spore germination of Penicillium chrysogenum bioprocesses in complex media
Source: Appl Microbiol Biotechnol. 2016 Aug 24;100(20):8923–30. doi: 10.1007/s00253-016-7787-y (PMC5035658; doi:10.1007/s00253-016-7787-y)
Supplement: Supplementary file 1 — (PDF 69 kb) [file 253_2016_7787_MOESM1_ESM.pdf]

Applied Microbiology and Biotechnology

At-line determining spore germination of *Penicillium chrysogenum* bioprocesses in complex media

Daniela Ehgartner[1,2], Jens Fricke[1,2], Andreas Schröder[1,2] and Christoph Herwig[1,2]*

*to whom the correspondence should be addressed to

[1] CD Laboratory on Mechanistic and Physiological Methods for Improved Bioprocesses, Technical University, Gumpendorferstrasse 1a/166, 1060 Vienna, Austria

[2] Research Area Biochemical Engineering, Institute of Chemical Engineering, Vienna University of Technology, Gumpendorferstrasse 1a/166, 1060 Vienna, Austria

Corresponding author:

christoph.herwig@tuwien.ac.at

Tel (Office): +43 1 58801 166400
Fax: +43 1 58801 166980

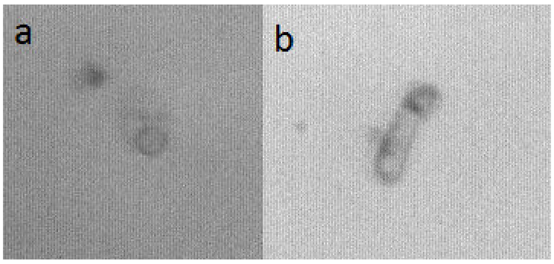

Fig. S1 Pictures of spores taken in the flow cell of the CytoSense. a) A non-germinated spore and a medium particle, b) a germinated spore going upright through the cell
